# Program of All-Inclusive Care for the Elderly (PACE) versus Other Programs: A Scoping Review of Health Outcomes

**DOI:** 10.3390/geriatrics7020031

**Published:** 2022-03-12

**Authors:** Daniel Arku, Mariana Felix, Terri Warholak, David R. Axon

**Affiliations:** 1 Department of Pharmacy Practice and Science, College of Pharmacy, The University of Arizona, 1295 North Martin Avenue, Tucson, AZ 85721, USA; arku@pharmacy.arizona.edu (D.A.); marianafelix@email.arizona.edu (M.F.); warholak@pharmacy.arizona.edu (T.W.); 2 Mel and Enid Zuckerman College of Public Health, The University of Arizona, 1295 North Martin Avenue, Tucson, AZ 85724, USA; 3 Center for Health Outcomes & Pharmacoeconomic Research (HOPE Center), College of Pharmacy, The University of Arizona, 1295 North Martin Avenue, Tucson, AZ 85721, USA

**Keywords:** geriatrics, community-based care, PACE, models of care, outcomes

## Abstract

The Program of All-Inclusive Care for the Elderly (PACE) provides comprehensive health and social services to community-dwelling older United States (US) adults. However, little is known about how PACE outcomes compare to similar caregiving programs. This scoping review searched nine databases to identify studies that compared economic, clinical, or humanistic outcomes of PACE to other caregiving programs in the US. Two reviewers independently screened and extracted data from relevant articles and resolved discrepancies through consensus. From the 724 articles identified, six studies were included. Example study outcomes included: limitations and needs, survival and mortality, healthcare utilization, and economic outcomes. In conclusion, there are few published comparisons of PACE outcomes versus other caregiving programs for older US adults, and identified studies indicate mixed results. Further studies are needed to compare PACE outcomes to other programs so that policymakers are well informed to manage and optimize health outcomes for the growing US older adult population.

## 1. Introduction

Older adults account for an increasingly large proportion of the United States (US) population. The proportion of US adults aged 65 and older is expected to nearly double from 52 million in 2018 to 95 million by 2060 [1]. Older adults are the fastest-growing age group and therefore account for the majority of complex patients with multiple chronic conditions and significant social and health needs [2]. A recent study demonstrated that while 95% of patients with complex medical needs have standard access to care, 58% do not have a care coordinator to help them navigate the system, 37% feel lonely and isolated, and 62% experience stress over their ability to afford housing or healthy food. In addition, 47% of patients with complex medical needs have visited the emergency department for an illness that could have been treated in a doctor’s office or clinic [3]. There is therefore a need for services that help the increasing population of older adults with their health and social care needs. The increase in older adults with healthcare needs also means that Social Security and Medicare expenses will increase from a combined 9.3% of the gross domestic product in 2021 to 11.8% by 2035 [4]. As a result, care provision services for the elderly that promote healthy living and support independence are emerging. Examples of these services include independent living facilities, assisted living, skilled nursing facilities, continuous care retirement communities, and the Program of All-Inclusive Care for the Elderly (PACE).

PACE aims to deliver comprehensive medical and social care from an interdisciplinary team of healthcare providers to community-dwelling elderly individuals that allows participants to remain living in their homes rather than receiving care in a nursing home [5]. Individuals are eligible for PACE if they are aged 55 years or older, live in an area serviced by a PACE organization, are eligible for nursing home care, and can safely live in their own community [5]. Most PACE participants are dual-eligible for Medicare and Medicaid services [5]. PACE organizations can provide all necessary healthcare services to beneficiaries using a capped financing model [5]. Research suggests that participation in PACE is associated with improved care quality, less mortality, preservation of function, fewer unmet assistance needs, greater participant and caregiver satisfaction, less hospital and nursing home utilization, and lower Medicare costs [6]. However, data that compares PACE outcomes to other similar programs for older adults have not been synthesized. This is important to know given the anticipated growth in the need for such services as the older adult population increases. The first step in addressing this literature gap is to identify studies that have compared PACE with other similar programs for older adults, and to summarize the types of outcomes reported in these studies. This information will allow researchers to identify what is already known about how PACE compares to other similar programs for older adults and identify areas where further enquiries are needed to optimize health outcomes for older adults.

The purpose of this study was to identify and describe the various health outcomes of individuals enrolled in PACE compared to older adults enrolled in other similar programs.

## 2. Methods

This scoping review was conducted following the Preferred Reporting Items for Systematic Reviews and Meta-analysis extension for Scoping Reviews (PRISMA-ScR) and grounded in Arksey and O’Malley’s five-stage framework, which utilizes a stringent process of transparency, allowing duplication of the search strategy and improving the reliability of the study findings [7,8]. The five stages of the framework used in this literature review on evaluating health outcomes of participants enrolled in PACE and elderly participants enrolled in other caregiving programs include: (1) identifying the initial research questions; (2) identifying relevant studies; (3) study selection; (4) data charting and collating; and (5) summarizing and reporting findings.

### 2.1. Identifying the Initial Research Question

The goal of this scoping literature review was to identify and describe the outcomes of participants enrolled in PACE versus other similar programs. A scoping review was deemed the most appropriate type of literature review since comparator programs and outcomes reported in the literature were unknown. This review focused on identifying comparative studies to compare the economic, clinical, and humanistic outcomes (ECHO model) of PACE to other similar programs [9]. The following items were used to develop the initial research question: (1) What is known about the PACE model and models of care for community dwelling adults? (2) What other comparable caregiving programs for the elderly exists besides PACE? (3) What outcomes (economic, clinical, and humanistic) are commonly reported from PACE and other comparable models of care for the elderly? (4) What considerations are given when evaluating outcomes from the PACE model and other comparable models of care for the elderly?

### 2.2. Identifying Relevant Studies

Key concepts and search terms were developed to capture literature that related to the PACE model and other comparable models of care for older adults. All relevant articles published between 1 January 1997 (date PACE was established under the Balanced Budget Act of 1997 as a permanent part of the Medicare program and an option under state Medicaid program) [10] and 12 March 2021 (final search date) were screened.

Studies were included if they were written in English, set in the US, involved at least two groups of participants (one group enrolled in PACE and another group in a comparable program), and reported on any economic, clinical, or humanistic outcome(s). Studies were excluded if they were not original reports (e.g., reviews, editorials, letters, commentaries, or duplications), did not have the necessary comparator groups, or were not evaluating outcomes of PACE participants versus another comparable model of care for older adults.

Nine electronic databases were searched for peer-reviewed articles: Medline PubMed, Scopus, Embase, Ovid Medline/Ovid Embase, Cochrane Library, CINAHL, PsycInfo, Business Source Ultimate, and ABI. The reference lists of identified articles in Google Scholar were also searched to identify any additional studies. The following search terms were used in PubMed and adapted for use in the other databases: “program” [All Fields] OR “program’s” [All Fields] OR “programe” [All Fields] OR “programed” [All Fields] OR “programes” [All Fields] OR “programing” [All Fields] OR “programmability” [All Fields] OR “programmable” [All Fields] OR “programmable” [All Fields] OR “programme” [All Fields] OR “programme’s” [All Fields] OR “programmed” [All Fields] OR “programmer” [All Fields] OR “programmer’s” [All Fields] OR “programmers” [All Fields] OR “programmes” [All Fields] OR “programming” [All Fields] OR “programmings” [All Fields] OR “programs” [All Fields] Elderly: “aged” [MeSH Terms] OR “aged” [All Fields] OR “elderly” [All Fields] OR “elderlies” [All Fields] OR “elderly’s” [All Fields] OR “elderlys” [All Fields].

### 2.3. Study Selection

**Figure 1:**
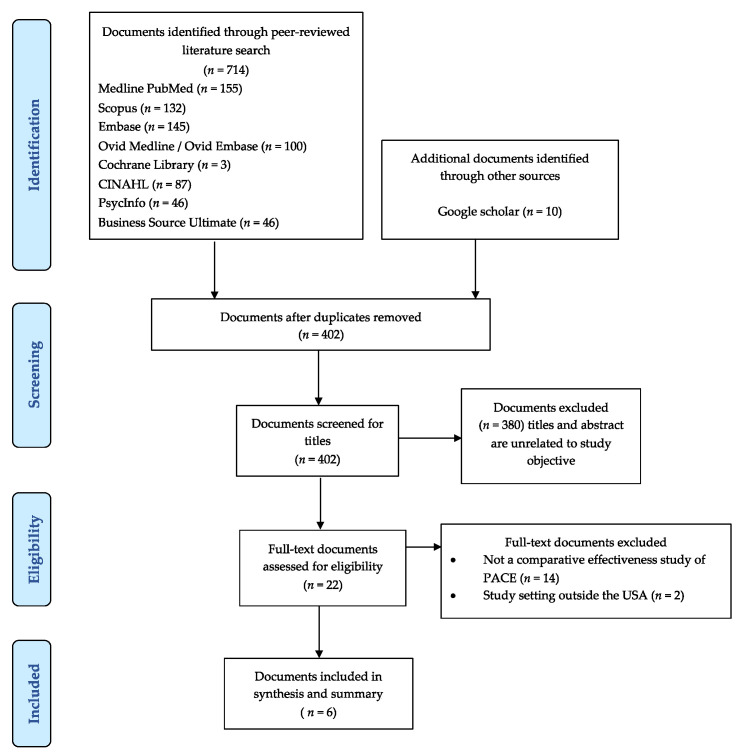
Flowchart of the study inclusions and exclusions of articles.

The Preferred Reporting Items for Systematic Reviews and Meta-analysis extension for Scoping Reviews (PRISMA-ScR) were followed for the study selection process (Figure 1) [7]. Full text versions of identified articles were imported into Mendeley. Two independent reviewers (DA and MF) evaluated the titles, abstracts, and full text of all articles identified for potential inclusion in the study. The reviewers met at each stage to ensure consistency of the results. Disagreements were settled through discussion with a third reviewer (DRA) as necessary until consensus was reached.

### 2.4. Data Charting and Collation

Data from each article were extracted using a data-charting form, which included: study author, year, study design, study duration, sample size in each group, intervention, comparator, patient age, and patient gender. The data charting form also collected all relevant aspects of any outcome, such as health care resource utilization, various measures of limitations with activities of daily living (ADL) and instrumental ADL, survival, and mortality rates of participants in each program with their respective *p*-values for statistical significance. Both reviewers independently charted the data, discussed the results, and updated the data-charting form as it was refined. Data charting was completed over two months, ending in April 2021.

### 2.5. Summarizing and Reporting Findings

Finally, per Arksey and O’Malley’s five-stage framework for scoping reviews [8], findings from the review were summarized and reported. First, a summary of relevant characteristics of each included study were reported in Table 1. Second, the outcomes assessed in PACE models and how they compared with other programs for each study were reported in Table 2, Table 3 and Table 4.

### 2.6. Risk of Bias Assessment

All studies included in the scoping review were observational studies, hence risk of bias was assessed using the Risk of Bias in Non-Randomized Studies—of Interventions (ROBINS-I) tool [11]. The tool assessed seven bias domains: (1) confounding; (2) selection of participants into the study; (3) classification of interventions; (4) deviations from intended interventions; (5) missing data; (6) measurement of outcomes; and (7) selection of the reported result. These biases could be reported as having a low, moderate, serious, or critical risk of bias [11]. Two investigators (DA and MF) independently assessed the risk of bias for each study and scored each domain. The reviewers then met to resolve differences until consensus was reached with the help of a third reviewer (DRA) as necessary.

## 3. Results

### 3.1. Identified Studies

A total of 724 articles were identified and retrieved from Medline PubMed (155 articles), Scopus (132 articles), Embase (145 articles), Ovid Medline/Ovid Embase (100 articles) Cochrane Library (3 articles), CINAHL (87 articles), PsycInfo (46 articles), Business Source Ultimate (46 articles), and Google Scholar (10 articles). No relevant articles were identified in the ABI inform database. After excluding duplicates, 402 records were screened, from which 380 were excluded since the titles and/or abstracts were unrelated to the study objective or were not conducted in the US. Therefore, 22 records were reviewed in full, of which six met the eligibility criteria and were included in this scoping review [6,12,13,14,15,16]. The majority of the articles that were excluded did not compare the health outcomes of PACE participants with participants in other caregiving programs for older adults (Figure 1).

### 3.2. Characteristics of Studies

Three studies were retrospective cohort, two were prospective cohort, and one was a cross-sectional study. The majority of study participants were female (>65%) in both PACE and comparator groups. The mean age of participants was >60 years (Table 1).

### 3.3. Description of Programs Included in the Review

Among the six articles included in this review, two articles assessed the limitations of daily living and healthcare-resource utilization among: (1) enrollees in PACE and Medicaid-only-managed long-term care (MMLTC) [12] and (2) enrollees in PACE and a Visiting Nurse Service (VNS) Choice Program (VCP) [13]. One article assessed limitations of daily living among PACE enrollees and participants in a Wisconsin Partnership Program (WPP) [14]. One article assessed healthcare-resource utilization and mortality rates among participants in a Veterans Affairs (VA) model with PACE, a VA-community partnership with PACE (VA + PACE), and VA as a sole provider of care (VA-Sole) [15]. Another assessed five-year survival rates among enrollees in PACE, a nursing home, and a waiver program for the elderly and disabled [16]. The remaining study assessed Medicaid attrition-adjusted one-year payment for enrollees in PACE, a nursing home, and a waiver program for the elderly and disabled [6].

The MMLTC model is used for integrating care to coordinate the delivery, but not the financing, of acute and long-term care services. This model places emphasis on home-based personal care [12]. The VCP is a partially integrated model capitated to deliver long-term care services only, by looking at health outcomes over 18 months of enrolment [13]. WPP is a variant of PACE that allows enrollees to remain with their own primary care physician and to make substantially less use of day care by using more care at home [14]. VA + PACE is a program where the VA partners with a local PACE community provider to share care responsibilities [15]. VA-Sole is a model where the VA is the sole provider of all healthcare needs, such as hospital, specialty, and nursing home, among others [15]. The nursing home model provides nursing, therapy, and personal care services to individuals who do not require acute hospital care, but whose mental or physical condition requires services that are above the level of room and board and can be made available through licensed, certified, and contracted institutional facilities [6]. The aged and disabled waiver program is a Medicaid community-based waiver program available for adults qualifying for Medicaid and certified as nursing home eligible but who prefer to receive services in the community [16].

### 3.4. Study Outcomes

Study outcomes were organized into one of four groups that included: (1) activities-of-daily-living (ADL) limitations, instrumental-activities-of-daily-living (IADL) limitations, and unmet needs; (2) healthcare resource use; (3) clinical and survival outcomes; and (4) economic outcomes.

### 3.5. ADL, IADL, and Unmet Need Outcomes

Three studies assessed ADL and IADL limitations among PACE participants and participants in comparator programs. In one study, a greater percentage of PACE participants reported needing help with most ADL and IADL limitations compared to WPP participants [14]. The same study also reported no significant differences in the proportion of PACE participants and WPP participants with unmet needs [14]. In a second study, the mean number of ADL limitations reported was lower among PACE participants than that of MMLTC participants after 12 months, yet the mean number of IADL limitations reported was higher among PACE participants compared to MMLTC participants [12]. In a third study, more PACE participants reported a decline in ADL compared to VCP participants after 18 months and fewer PACE participants reported improvement in ADL limitations compared to VCP participants [13]. In the same study, fewer PACE participants reported a decline in IADL limitations than VCP participants and fewer PACE participants reported an improvement in IADL limitations than VCP participants [13] (Table 2).

### 3.6. Healthcare Resource Use Outcomes

Three studies assessed healthcare resource use among PACE participants and participants in comparator programs [12,13,15]. In a 12-month resource utilization study, home- and community-based services (HCBS) utilization per member per month was greater among PACE participants than MMLTC participants, while home-delivered healthcare service utilization was typically lower among PACE participants than MMLTC participants [12]. There were more adult day center visits per member per month and more nursing home users among PACE participants than among MMLTC participants, yet fewer hospital users, a lower proportion of days in the hospital, and a lower mean length of stay (LOS) in the hospital among PACE participants than among MMLTC participants [12]. In another study, there were more nursing home users and a greater proportion of days in nursing homes among PACE participants than among VCP participants, yet fewer hospital users, a lower proportion of days in the hospital, and a lower mean LOS in the hospital among PACE participants than among VCP participants [13]. In a further 12-month resource utilization study, data on inpatient admissions, nursing home admissions, outpatient visits, home care visits, and adult day care visits were reported for three programs: PACE, Veteran’s Affairs, and a Veteran’s Affairs partnership with PACE; however, no statistical differences were reported between the programs [15] (Table 3).

### 3.7. Clinical and Survival Outcomes

Four studies reported clinical and survival outcomes [13,14,15,16]. One study reported measures of pain, discomfort, and depression among PACE participants and WPP participants, but there were no statistically significant differences reported between the two programs [14]. In another study, the proportion of deaths was greater among PACE participants than VCP participants [13]. Another study reported the proportion of individuals who survived and the proportion who died for three programs: PACE, Veteran’s Affairs, and a Veteran’s Affairs partnership with PACE; however, no statistical differences were reported between the programs [15]. One further study reported a five-year risk-adjusted survival rate for those in PACE, longer than for those in a nursing home and those in a waiver program for the elderly and disabled [16] (Table 4).

### 3.8. Economic Outcomes

One study reported economic outcomes. In that study, the average Medicaid attrition-adjusted one-year payment reported for enrollees in PACE, a nursing home, and a waiver program for the elderly and disabled, were USD 36,620 (95% CI = USD 35,662–USD 37,580), USD 77,945, and USD 4177 respectively for the 2005 fiscal year. Significance tests were not reported [6].

### 3.9. Risk of Bias in Included Studies

Most studies were primarily descriptive; thus, it was challenging to fully assess the risk of bias. Therefore, many risk-of-bias domains were not applicable, were unclear, or had no information and thus were marked as “no information”. Bias due to confounding was considered high risk in both studies by Wieland (2010 and 2013) [6,16]. However, Wieland (2010) used an established mortality risk index to address cohort risk imbalances and assessed 5-year survival rate based on these risk stratifications [16]. Weiland (2013) also partitioned individuals based on their similarities to clinical profiles by assigning grades of membership to the models of care and using a general multivariate procedure for analyzing high-dimensional discrete response data based on maximum likelihood principles to account for the different health needs associated with the different programs of long-term care assessed [6]. All studies had no information on missing data, thus the risk of bias due to missing data was marked as “no information” for all the studies. In all other instances, the risk of bias was considered low or moderate. Based on the ROBINS-I tool, the overall judgment for the risk of bias was low for two studies [6,16], moderate for two studies [12,13], and there was a lack of information to make an overall judgment for two studies [14,15] (Table 5).

## 4. Discussion

This scoping review adopted a systematic approach to identify and describe the comparative effectiveness studies of economic, clinical, and humanistic outcomes for PACE participants in comparison to other models of care for older adults from multiple databases. This review included six studies, which highlights the scarcity of comparative effectiveness studies in the literature regarding PACE and other long-term care models for older adults. This review also addresses the limited and mixed evidence suggesting better outcomes for PACE participants than participants in other programs of care for older adults. The six studies reported outcomes such as ADL limitations, IADL limitations, unmet needs, healthcare resource use, clinical and survival outcomes; and economic outcomes, which are discussed below.

ADL and IADL limitations accounted for the most outcomes identified, with three (50%) of the six studies reporting on these outcomes. Measuring functional disability among older adults according to elements of ADL and IADL limitations is an established concept [17,18,19,20,21], hence this approach is rational for assessing benefits associated with different models of care for older adults. These three studies reported mixed results for PACE participants relative to their respective comparators. For instance, Nadash (2013) [13] reported a significant improvement in the IADL and ADL limitations in PACE participants compared to VCP participants, and Nadash (2004) [12] reported significantly fewer ADL and IADL limitations in PACE participants compared to MMLTC participants, suggesting that PACE is superior to VCP and MMLTC for managing functional disability among older adults. However, Kane et al. (2002) [14] reported a significant proportion of PACE participants needed help with various measures of ADL and IADL limitations compared to WPP participants. Additionally, Kane et al. (2002) reported no significant difference with respect to the proportion of participants with unmet needs [14]. This represents patients with dependencies who needed help in the form of bathing, dressing, toileting, etc., but did not receive help [14]. Since other studies [22,23] have demonstrated the health consequences of having unmet caregiving needs, it is prudent to understand which care models effectively address functional needs to better assist community-dwelling older US adults.

In relation to healthcare-resource utilization, the current review showed that, on average, PACE participants had lower rates of hospital use [15], with shorter lengths of stay in hospitals (<6 days) within 12 months compared to other programs [12,13]. These findings are consistent with another study that showed that PACE participants had shorter hospital stays compared to non-PACE participants [24]. A retrospective study observed the outcomes of 61 PACE organizations. Program enrollment and hospital inpatient usage data were used to measure overall hospitalization and readmission rates. Rates of hospitalization, readmission, and potentially avoidable hospitalizations were lower for PACE enrollees than for comparable populations. This confirmed that the studies in our review are consistent with those from earlier studies [25]. However, the current review found higher rates of nursing home utilization per member per month for PACE participants compared to MMLTC participants during the first year of enrollment [12]. This finding is contrary to studies that showed a reduction in nursing home utilization among PACE participants compared to others [24,26].

Specific and all-cause mortality rates among older US adults is often assessed in studies regarding this population [27,28,29,30]. Three (50%) of the six studies included in the current review assessed mortality risk and the proportion of deaths in PACE versus other programs [13,15,16]. Wieland et al. (2010) reported PACE participants survived longer (4.2 years) than the 5-year median survival for those in a nursing home (2.3 years) and (3.5 years) in a waiver program [16]. These findings align with others that found PACE delivered favorable results with increased longevity and less institutionalization compared to a nursing home [24,31,32]. However, Nadash (2013) [13] reported that 20% of participants in PACE died compared to 10% in VCP after 18 months, although this may be because PACE participants were sicker than VCP participants and the risk of death for participants who received treatment for all causes was lower (HR = 0.55) in VCP compared to PACE. However, when propensity scores were used to match the groups, there was no significant difference between PACE and VCP groups [13]. Weaver et al. (2008) [15] found that 34% of PACE participants died compared to 28% of participants in the VA sole-provider model and 28% of participants in the VA + PACE model in a 36-month post-enrollment study, although the study did not report any significance tests. Thus it is not possible to know if the proportion who died in PACE was statistically greater than the other programs compared. A study by Meret (2011), evaluated the effects of PACE on hospital use over four 6-month intervals and a 2-year follow-up period [33]. The results showed that over the two-year follow-up period, the comparison group, which comprised of frail community-dwelling older adults selected through propensity score matching over a 2-year period, had a mortality rate of 24.9%, almost 5% higher than the PACE sample [33].

Only one study compared costs associated with PACE, a nursing home, and a waiver program. The average 2005 fiscal year Medicaid attrition-adjusted one-year payment for PACE participants was about seven times more expensive than those in the waiver program in the study and about twice lower than the cost of those in nursing homes, but with no test for significant difference reported [6]. This finding is consistent with other studies, where higher Medicaid costs were reported for PACE enrollees, but no significant difference in Medicare costs between PACE and matched home- and community-based services enrollees or those in nursing homes were observed [34,35,36]. Conversely, the findings are not consistent with a study that found costs for PACE participants were 16–38% lower than Medicare fee-for-service costs for a frail elderly population, and 5–15% lower than costs for comparable Medicaid beneficiaries [37]. However, these costs are now outdated and may not reflect costs for all services incurred due to the variability in the programs.

Findings from this scoping review highlight the need to promote more research around care models for older adults. As the US population ages and life expectancy continues to increase, the economic, clinical, and humanistic burden associated with caring for older adults will also increase. Thus, further research in this area is needed to equip health policymakers and interested stakeholders with the necessary information to manage and improve care for older adults.

This scoping review has some limitations. There were very few (*n* = 6) studies that met the eligibility criteria for this study, as most of the literature focused on evaluating the outcomes of PACE models without comparing outcomes with other non-PACE models of care. In addition, the limited amount of data for the limited number of outcomes meant a meta-analysis could not be performed to obtain a single summary estimate. The findings of the individual studies may have been influenced by the eligibility or selection of beneficiaries for the respective programs, and the different services offered by different programs, which may have influenced the results. Furthermore, many of the studies did not report *p*-values to ascertain if differences between groups were statistically different. Finally, most of the studies were primarily descriptive; as such, it was difficult to perform a thorough risk-of-bias assessment since most of the risk-of-bias assessment domains were either not applicable, were unclear, or had no information.

## 5. Conclusions

The results from this scoping review highlight the limited evidence in the literature comparing PACE to other programs. The six studies included in this review included ADL and IADL limitations, unmet health and social care needs, healthcare-resource utilization, clinical and survival outcomes, and one study reported economic outcomes. The limited literature does provide some evidence that PACE provides quality and cost-effective community-based care to older adults who would otherwise require a nursing home or other model of care, although some other programs also have their advantages. There is a need for additional robust comparative effectiveness studies of PACE and other care models for older adults to improve our understanding of health outcomes in this population.

## Figures and Tables

**Table 1:** Characteristics of studies included in the scoping review.

Study Authors, Year	Study Design	Study Duration (Days)	PACE (N)	Comparison (N)	Comparison	PACE Patient Age Years, Mean ± SD or %	PACE Female (%)	Comparison Patient Age Years, Mean ± SD or %	Comparison Female (%)
Kane et al., 2002	Cross sectional	730	322	304	Wisconsin Partnership Program (WPP)	80 ± NR	82.0	77 ± NR	74.0
Nadash, 2004	Retrospective Cohort	365	1382	1267	Medicaid-only-managed long-term care	79± NR	71.5	79 ± NR	72.5
Nadash, 2013	Retrospective Cohort	540	1535	1540	VNS CHOICE program (VCP)	≥65 ± NR	71.5	≥65 ± NR	72.9
Weaver et al., 2008	Prospective Cohort	1095	85	181	VA as a sole provider	76 (range; 56.1–93.2)	1.0	75 (range; 55.6–101.3)	4.0
				102	VA & PACE partnership with PACE			77 (range; 55.2–94.6)	6.0
Wieland et al., 2010	Prospective Cohort	1825	554	468	Nursing Home	77.2 ± 0.42	65.9	74.8 ± 0.51	63.3
				1018	Aged and disabled waiver program			74.5 ± 0.32	75.5
Wieland et al., 2013	Retrospective Cohort	4015	948	1357	Nursing Home	55–64 (10.6%) 65–74 (27.0%) 75–84 (38.6%) ≥85 (23.8%)	75.2	55–64 (18.7%) 65–74 (20.3%) 75–84 (38.3%) ≥85 (22.7%)	63.1
				1683	Aged and disabled waiver program			55–64 (18.8%) 65–74 (27.6%) 75–84 (34.6%) ≥85 (19.0%)	76.4

PACE = Program of All Inclusive Care for the Elderly. VA = Veteran’s Affairs. NR = not reported. SD = standard deviation. Wisconsin Partnership Program (WPP) is a variant of PACE that allows enrollees to remain with their own primary care physician and to make substantially less use of day care. Medicaid-only-managed long-term care is a model for integrating care to coordinate the delivery, but not the financing of acute and long-term care services. VNS CHOICE program (VCP) is a partially integrated model capitated to deliver long-term care services. VA as a sole provider is a model where the VA is the sole provider of all healthcare needs, such as hospital, specialty, nursing home, etc. VA & PACE partnership with PACE is a partnership between the VA and a local community PACE provider to share care responsibilities. Nursing home provides nursing, therapy, and personal care services to individuals who do not require acute hospital care but whose mental or physical condition requires services that are above the level of room and board and can be made available through licensed, certified, and contracted institutional facilities. Aged and disabled waiver program is a Medicaid community-based waiver program available for adults qualifying for Medicaid and certified as nursing home eligible but who prefer to receive services in the community.

**Table 2:** ADL, IADL, and unmet need outcomes reported in studies included in the scoping review.

Study Authors, Year	Outcomes	PACE	Comparison 1	*p*
Kane et al., 2002		PACE (%)	WPP (%)	
	Needs a little help or more with ADLs			
	Bathing	64	44	0.000
	Dressing	47	29	0.000
	Toileting	32	15	0.000
	Transferring	28	17	0.002
	Feeding	15	5	0.000
	Able to walk between rooms	79	84	ns
	Difficulty with IADLs			
	Shopping	74	63	0.003
	Using phone	38	28	0.014
	Doing light housework	67	53	0.000
	Preparing meals	75	59	0.000
	Using transportation	42	35	ns
	Taking medications	26	16	0.002
	Managing finances	75	53	0.000
	Arranging services	73	54	0.000
	Unmet Needs, % (patients with unmet needs/patients with dependency			
	Need help with bathing and did not receive	8	9	ns
	Not able to bathe	15	15	ns
	Need help with dressing and did not receive	9	16	ns
	Unable to put on clean clothes	5	10	ns
	Need help with toileting and did not receive	27	10	ns
	Wet or soiled because no help available	54	30	ns
	Had to wait 20 min or more wet/soiled	18	19	ns
	Need help transferring and did not receive	11	17	ns
	Fell because no help	6	6	ns
	Need help with eating and did not receive	4	29	ns
	Hungry because no help	4	7	ns
	Thirsty because no help	6	14	ns
Nadash, 2004		PACE	MMLTC Plan	
	Mean ADL ^i^ limitations	2.9	3.6	<0.0001
	Mean IADL ^i^ limitations	5.6	5.5	<0.0001
Nadash, 2013		PACE	VCP	
	Patients with decline in ADL ^ii^, %	27.05	23.69	0.0461
	Patients with no change in ADL ^ii^, %	21.18	21.90	0.6500
	Patients with improved ADL ^ii^, %	16.50	29.90	<0.0001
	Patients with decline in IADL ^ii^, %	11.03	13.68	0.0431
	Patients with no change in IADL ^ii^, %	48.79	45.29	0.0734
	Patients with improved IADL ^ii^, %	4.83	16.52	<0.0001

PACE = Program of All-inclusive Care for the Elderly. WPP = Wisconsin Partnership Program. MMLTC = Medicaid-only-managed long-term care. VCP = Visiting Nurse Service (VNS) Choice Program. ADL = activities of daily living. IADLs = instrumental activities of daily living. ns = nonsignificant. ^i^ I/ADLS scales were constructed in original study, with any need for assistance coded as ‘1′ and summed (range = 0–5). ^ii^ 18 months post-enrollment.

**Table 3:** Healthcare resource use outcomes reported in studies included in the scoping review.

Study Authors, Year	Outcomes	PACE	Comparison 1	Comparison 2	*p*
Nadash, 2004		PACE	MMLTC Plan		
	HCBS utilization per member per month ^i^				
	Nursing	8.42	2.20		<0.0001
	Nurse practitioner	0.93	0.05		<0.0001
	Social work	2.79	0.37		<0.0001
	Ancillary therapists	6.43	0.73		<0.0001
	Home-delivered services utilization per member per month ^i^				
	Nursing	1.16	2.20		<0.0001
	Nurse practitioner	0.07	0.05		0.0054
	Social work	0.35	0.37		<0.0001
	Ancillary therapists	0.24	0.73		<0.0001
	Adult day center visits per member per month ^i^	11.38	0.23		<0.0001
	Hospital users ^i^, %	33.7	35.2		0.0362
	Proportion of days in hospitals ^i^	1.0	2.0		<0.0001
	Mean LOS in hospitals ^i^ (days)	5.9	9.5		<0.0001
	Nursing home users ^i^, %	21.0	5.7		<0.0001
	Proportion of days in nursing homes ^i^	4.5	0.9		<0.0001
	Mean LOS in nursing homes ^i^ (days)	44.2	37.2		ns
Nadash, 2013		PACE	VCP		
	Hospital users ^i^, %	20.3	33.1		<0.0001
	Proportion of days in hospitals ^i^	0.6	2.3		<0.0001
	Mean LOS in hospitals ^i^ (days)	5.8	9.7		<0.0001
	Nursing home users ^i^, %	13.3	7.2		<0.0001
	Proportion of days in nursing homes ^i^	2.3	1.3		<0.0001
	Mean LOS in nursing homes ^i^ (days)	36.4	42.8		0.2767
	Patients discharged ^ii^, %	13.26	13.98		0.5915
Weaver et al., 2008		PACE	VA-Sole	VA+PACE	
	Patients with inpatient admissions, % ^i^	35	49	41	
	Inpatient admissions/patient, mean ± SD ^i^	0.56 ± 3.0	1.12 ± 5.3	0.68 ± 3.1	NR
	Total inpatient days/patient, mean ± SD ^i^	2.07 ± 14.7	8.55 ± 57.9	2.59 ± 15.0	NR
	Patients with nursing home admissions, % ^i^	38	26	40	NR
	Total nursing home admissions, mean ± SD ^i^	0.59 ± 2.9	0.41 ± 2.8	0.87 ± 3.9	NR
	Nursing home days/patient, mean ± SD ^i^	12.56 ± 127.9	10.96 ± 124.6	25.1 ± 09.3	NR
	Patients with outpatient clinic visits, % ^i^	100	97	100	NR
	Outpatient clinic visit/patient, mean ± SD ^i^	39.48 ± 74.8	23.45 ± 45.6	39.17 ± 5.4	NR
	Patients with home care visits, % ^i^	93	42	91	NR
	Number of home care visits/patient, mean ± SD ^i^	7.70 ± 21.9	16.46 ± 108.7	8.15 ± 25.9	NR
	Patients with adult day healthcare use, % ^i^	100	54	100	NR
	Adult day healthcare visits/patient, days, mean ± SD ^i^	165.87 ± 220.7	14.41 ± 95.4	120.5 ± 227.1	NR

PACE = Program of All-inclusive Care for the Elderly. MMLTC = Medicaid-only-managed long-term care. HCBS = home- and community-based services. LOS = Length of stay. VCP = Visiting Nurse Service (VNS) Choice Program. VA-Sole = Veteran’s Affairs as sole provider. VA+PACE = Veteran’s Affairs partnership with PACE. SD = standard deviation. ns = nonsignificant. NR = not reported. Ancillary therapists include occupational, physical, and speech therapists. ^i^ 12 months utilization (visits) post-enrollment. ^ii^ 18 months post-enrollment.

**Table 4:** Clinical and survival outcomes reported in studies included in the scoping review.

Study Authors, Year	Outcomes	PACE	Comparison 1	Comparison 2	*p*
Kane et al., 2002	Dependency/Discomfort	PACE (%)	WPP (%)		
	Pain/discomfort moderate/severe ^i^	44	49		ns
	Pain interferes with normal activity some/most of time ^i^	50	59		ns
	Very satisfied with pain control	91	91		ns
	Depression > 5 on GDS ^i^	15	18		ns
Nadash, 2013		PACE	VCP		
	Deaths ^ii^, %	22.05	10.01		<0.0001
	Risk of death (for participants who were treated); HR	ref	0.55		95% CI 0.26–1.22
Weaver et al., 2008		PACE	VA-Sole	VA + PACE	
	Deaths, *n* (%) ^iii^	29 (34)	52 (28)	28 (28)	NR
	Survived, *n* (%) ^iii^	37 (66)	113 (62)	51 (50)	NR
Wieland et al., 2010		PACE	Nursing Home	Waiver Program	
	Five-year median survival (years) ^iv^	4.2	2.3	3.5	0.015

PACE = Program of All-inclusive Care for the Elderly. WPP = Wisconsin Partnership Program. VCP = Visiting Nurse Service (VNS) Choice Program. VA-Sole = Veteran’s Affairs as sole provider. VA+PACE = Veteran’s Affairs partnership with PACE. GDS = Geriatric Depression Scale. ns = nonsignificant. NR = not reported. ^i^ based on patient report only. ^ii^ 18 months post-enrollment. ^iii^ 36 months after enrollment. ^iv^ risk-adjusted. HR = hazard ratio. CI = confidence interval. Ref = reference group.

**Table 5:** Risk of bias assessment for included observational studies.

Study Authors, Year	Bias Due to Confounding	Bias in Selection of Participants into the Study	Bias in Classification of Interventions	Bias Due to Deviations from Intended Interventions	Bias Due to Missing Data	Bias in Measurement of Outcomes	Bias in Selection of the Reported Results	Overall Risk of Bias Judgment
Kane et al., 2002	No information	No information	Low	No information	No information	Moderate	No information	No information
Nadash, 2004	Moderate	Low	Low	No information	Moderate	Moderate	No information	Moderate
Nadash, 2013	Low	Low	Low	No information	No information	Moderate	No information	Moderate
Weaver et al., 2008	Moderate	Moderate	Moderate	No information	No information	Moderate	No information	No information
Wieland et al., 2010	High	Low	Low	Low	No information	Low	Low	Low
Wieland et al., 2013	High	Low	Low	Low	No information	Low	No information	Low

## Data Availability

Not applicable.

## References

[1] Older People Projected to Outnumber Children for First Time in U.S. History. https://www.census.gov/newsroom/press-releases/2018/cb18-41-population-projections.html.

[2] Let’s Work Together to Improve Care for Older Adults with Complex Needs. https://www.healthaffairs.org/do/10.1377/hblog20170214.058749/full/.

[3] How High-Need Patients Experience Health Care in the United States. https://www.commonwealthfund.org/sites/default/files/documents/___media_files_publications_issue_brief_2016_dec_1919_ryan_high_need_patient_experience_hnhc_survey_ib_v2.pdf.

[4] A Summary of the 2021 Annual Reports. www.ssa.gov/oact/TRSUM/.

[5] Medicaid.gov Program of All-Inclusive Care for the Elderly. https://www.medicaid.gov/medicaid/long-term-services-supports/program-all-inclusive-care-elderly/index.html.

[6] Wieland D., Kinosian B., Stallard E., Boland R. (2013). Does Medicaid pay more to a program of all-inclusive care for the elderly (PACE) than for fee-for-service long-term care?. J. Gerontol. A Biol. Sci. Med. Sci..

[7] Tricco A.C., Lillie E., Zarin W., O’Brien K.K., Colquhoun H., Levac D., Moher D., Peters M.D.J., Horsley T., Weeks L. (2018). PRISMA extension for scoping reviews (PRISMA-ScR): Checklist and explanation. Ann. Intern. Med..

[8] Arksey H., O’Malley L. (2005). Scoping studies: Towards a methodological framework. Int. J. Soc. Res. Methodol..

[9] Kozma C.M., Reeder C.E., Schulz R.M. (1993). Economic, clinical, and humanistic outcomes: A planning model for pharmacoeconomic research. Clin. Ther..

[10] Centers for Medicare & Medicaid Services Program of All-Inclusive Care for the Elderly. https://www.cms.gov/Regulations-and-Guidance/Guidance/Manuals/Downloads/pace111c01.pdf.

[11] Sterne J.A., Hernán M.A., Reeves B.C., Savović J., Berkman N.D., Viswanathan M., Henry D., Altman D.G., Ansari M.T., Boutron I. (2016). ROBINS-I: A tool for assessing risk of bas in non-randomized studies of interventions. BMJ.

[12] Nadash P. (2004). Two models of managed long-term care: Comparing PACE with a Medicaid-only plan. Gerontologist.

[13] Nadash P. (2013). Comparing PACE with a Medicaid-only managed long-term care plan: Health outcomes over time. World Med. Health Policy.

[14] Kane R.L., Homyak P., Bershadsky B. (2002). Consumer reactions to the Wisconsin Partnership Program and its parent, the Program for All-Inclusive Care of the Elderly (PACE). Gerontologist.

[15] Weaver F.M., Hickey E.C., Hughes S.L., Parker V., Fortunato D., Rose J., Cohen S., Robbins L., Orr W., Priefer B. (2008). Providing all-inclusive care for frail elderly veterans: Evaluation of three models of care. J. Am. Geriatr. Soc..

[16] Wieland D., Boland R., Baskins J., Kinosian B. (2010). Five-year survival in a program of all-inclusive care for elderly compared with alternative institutional and home-and community-based care. J. Gerontol. A Biol. Sci. Med. Sci..

[17] Katz S., Akpom C.A. (1976). A measure of primary sociobiological functions. Int. J. Health Serv..

[18] Lawton M.P., Brody E.M. (1969). Assessment of older people: Self-maintaining and instrumental activities of daily living. Gerontologist.

[19] Millán-Calenti J.C., Tubío J., Pita-Fernández S., Gonzalez-Abraldes I., Lorenzo T., Fernandez-Arruty T., Maseda A. (2010). Prevalence of functional disability in activities of daily living (ADL), instrumental activities of daily living (IADL) and associated factors, as predictors of morbidity and mortality. Arch. Gerontol. Geriatr..

[20] Norström T., Thorslund M. (1991). The structure of IADL and ADL measures: Some findings from a Swedish study. Age Ageing.

[21] Freedman V.A., Martin L.G., Schoeni R.F. (2002). Recent trends in disability and functioning among older adults in the United States: A systematic review. JAMA.

[22] Depalma G., Xu H., Covinsky K.E., Craig B.A., Stallard E., Thomas J., Sands L.P. (2013). Hospital readmission among older adults who return home with unmet need for ADL disability. Gerontologist.

[23] Kelley A.S., Ettner S.L., Morrison R.S., Du Q., Sarkisian C.A. (2012). Disability and decline in physical function associated with hospital use at end of life. J. Gen. Intern. Med..

[24] The Impact of PACE on Participant Outcomes. https://www.cms.gov/Research-Statistics-Data-and-Systems/Statistics-Trends-and-Reports/Reports/Downloads/Chatterji_1998_6.pdf.

[25] Segelman M., Szydlowski J., Kinosian B., McNabney M., Raziano D.B., Eng C., van Reenen C., Temkin-Greener H. (2014). Hospitalizations in the Program of All-Inclusive Care for the Elderly. J. Am. Geriatr. Soc..

[26] Friedman S.M., Steinwachs D.M., Rathouz P.J., Burton L.C., Mukamel D.B. (2005). Characteristics predicting nursing home admission in the program of all-inclusive care for elderly people. Gerontologist.

[27] Bergen G., Stevens M.R., Burns E.R. (2016). Falls and fall injuries among adults aged ≥65 years—United States, 2014. MMWR Morb Mortal Wkly Rep..

[28] Cheng X., Wu Y., Yao J., Schwebel D.C., Hu G. (2016). Mortality from unspecified unintentional injury among individuals aged 65 years and older by US state, 1999-2013. Int. J. Environ. Res. Public Health.

[29] Tan E.J., Lui L., Eng C., Jha A.K., Covinsky K.E. (2003). Differences in mortality of black and white patients enrolled in the program of all-inclusive care for the elderly. J. Am. Geriatr. Soc..

[30] Schulz R., Beach S.R., Ives D.G., Martire L.M., Ariyo A.A., Kop W.J. (2000). Association between depression and mortality in older adults: The cardiovascular health study. Arch. Intern. Med..

[31] Effect of PACE on Costs, Nursing Home Admissions, and Mortality: 2006–2011. https://aspe.hhs.gov/reports/effect-pace-costs-nursing-home-admissions-mortality-2006-2011-0.

[32] Petigara T., Anderson G. (2009). Program of All-Inclusive Care for the Elderly. Health Policy Monitor.

[33] Meret-Hanke L.A. (2011). Effects of the Program of All-inclusive Care for the Elderly on hospital use. Gerontologist.

[34] The Effects of PACE on Medicare and Medicaid Expenditures. https://www.cms.gov/Research-Statistics-Data-and-Systems/Statistics-Trends-and-Reports/Reports/downloads/Foster_PACE_2009.pdf.

[35] Evaluation of the Program of All-Inclusive Care for the Elderly: A Comparison of the PACE Capitation Rates to Projected Costs in the First Year of Enrollment. https://www.cms.gov/Research-Statistics-Data-and-Systems/Statistics-Trends-and-Reports/Reports/Downloads/White_2000_6.pdf.

[36] PACE: An Evaluation. https://www.dshs.wa.gov/sites/default/files/rda/reports/research-8-26.pdf.

[37] White A.J. (1998). The Effect of PACE on Costs to Medicare: A Comparison of Medicare Capitation Rates to Projected Costs in the Absence of PACE.

